# Machine learning and bioinformatics analysis to identify autophagy-related biomarkers in peripheral blood for rheumatoid arthritis

**DOI:** 10.3389/fgene.2023.1238407

**Published:** 2023-09-13

**Authors:** Guoqi Dong, Hui Gao, Yingqi Chen, Huayuan Yang

**Affiliations:** School of Acupuncture-Moxibustion and Tuina, Shanghai University of Traditional Chinese Medicine, Shanghai, China

**Keywords:** rheumatoid arthritis, machine learning, autophagy, immune infiltration, diagnostic model

## Abstract

**Background:** Although rheumatoid arthritis (RA) is a common autoimmune disease, the precise pathogenesis of the disease remains unclear. Recent research has unraveled the role of autophagy in the development of RA. This research aims to explore autophagy-related diagnostic biomarkers in the peripheral blood of RA patients.

**Methods:** The gene expression profiles of GSE17755 were retrieved from the gene expression ontology (GEO) database. Differentially expressed autophagy-related genes (DE-ARGs) were identified for the subsequent research by inserting autophagy-related genes and differentially expressed genes (DEGs). Three machine learning algorithms, including random forest, support vector machine recursive feature elimination (SVM-RFE), and least absolute shrinkage and selection operator (LASSO), were employed to identify diagnostic biomarkers. A nomogram model was constructed to assess the diagnostic value of the biomarkers. The CIBERSORT algorithm was performed to investigate the correlation of the diagnostic biomarkers with immune cells and immune factors. Finally, the diagnostic efficacy and differential expression trend of diagnostic biomarkers were validated in multiple cohorts containing different tissues and diseases.

**Results:** In this study, 25 DE-ARGs were identified between RA and healthy individuals. In addition to “macroautophagy” and “autophagy-animal,” DE-ARGs were also associated with several types of programmed cell death and immune-related pathways according to GO and KEGG analysis. Three diagnostic biomarkers, EEF2, HSP90AB1 and TNFSF10, were identified by the random forest, SVM-RFE, and LASSO. The nomogram model demonstrated excellent diagnostic value in GSE17755 (AUC = 0.995, 95% CI: 0.988–0.999). Furthermore, immune infiltration analysis showed a remarkable association between EEF2, HSP90AB1, and TNFSF10 expression with various immune cells and immune factors. The three diagnostic biomarkers also exhibited good diagnostic efficacy and demonstrated the same trend of differential expression in multiple validation cohorts.

**Conclusion:** This study identified autophagy-related diagnostic biomarkers based on three machine learning algorithms, providing promising targets for the diagnosis and treatment of RA.

## 1 Introduction

As the most prevalent autoimmune and inflammatory arthritis, rheumatoid arthritis (RA) is characterized by joint pain and swelling (Liu et al., 2021), which strongly compromises the function of limbs and quality of life. The global incidence of RA was estimated to be between 0.5% and 1% (Catrina et al., 2017). Over the past few years, RA has emerged as a major cause contributing to disability (Huang et al., 2022). Although our understanding of the pathogenesis of the disease has evolved tremendously, patients with RA continue to endure physical damage and psychological distress. Therefore, it is essential to determine quantitative and objective biomarkers of RA.

Autophagy is critical for disassembling dysfunctional and unnecessary intracellular components as a conserved process of protein degradation (Baehrecke, 2005). It is directly linked to the progression of autoimmune diseases (Deretic and Levine, 2018). As a common form of programmed cell death, autophagy has been speculated as a candidate innovative therapeutic target for RA (Zhao et al., 2021). Autophagy may influence the progression and recovery of RA by affecting bone metabolism disorders in osteoblasts and osteoclasts (Wang et al., 2020). It has been found that the knockdown of P2X7R may decrease the expression of autophagy-related proteins, leading to the suppression of osteoclast differentiation (Ma et al., 2022). Previous research also reported that the PI3K/AKT/mTOR pathway inhibition promotes fibroblast-like synoviocytes (FLSs) autophagy, which suppresses the invasion of FLSs and exerts anti-arthritic effects (Yang et al., 2022). Furthermore, autophagy of immune cells in peripheral blood may be involved in the pathogenesis of RA. It has been observed that the level of autophagosomes in the circulating immune cells of RA patients is significantly higher than that of normal individuals (Chen et al., 2018). Meanwhile, patients with RA who responded to anti-TNF drugs had significantly lower LC3-II levels of peripheral monocyte than non-responders (Vomero et al., 2019). Therefore, autophagy-related genes in peripheral blood may be diagnostic biomarkers and promising therapeutic targets for RA.

**FIGURE 1:**
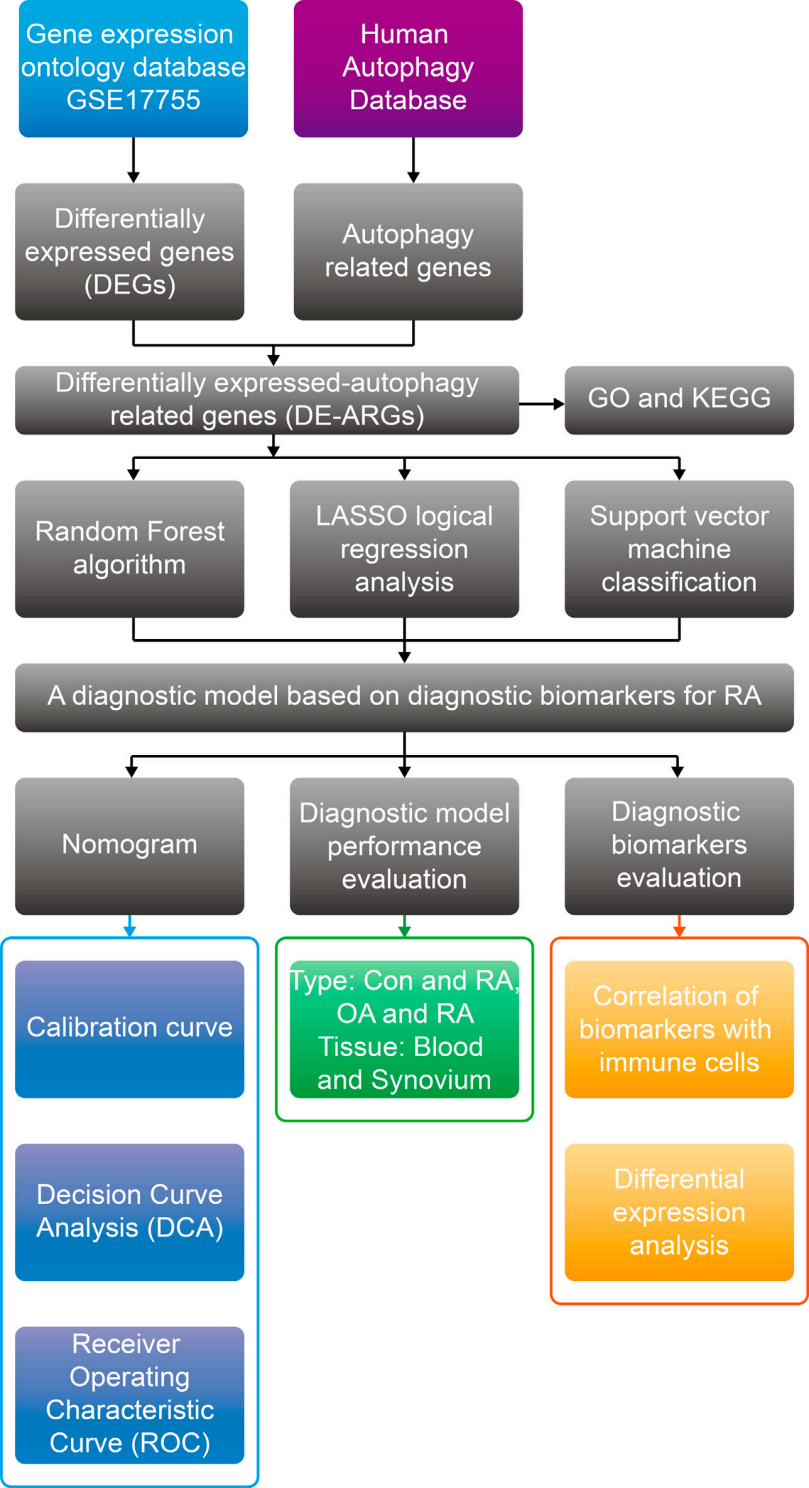
Overview of the research procedure of this study.

The study of RA pathology has utilized gene chip technology, which has led to the discovery of diagnostic biomarkers for RA through programmed death-related genes (Fan et al., 2023; Jiang et al., 2023; Li et al., 2023). This study screened DE-ARGs between normal and RA samples by differential analysis based on abundant public datasets. Meanwhile, machine learning algorithms, including random forest, LASSO and SVM-RFE, were carried out to identify diagnostic biomarkers related to autophagy. The correlation between diagnostic biomarkers and infiltrating immune cells was determined in RA samples by the CIBERSORT algorithm. To ensure the reproducibility and robustness of the results, we evaluated the diagnostic efficacy of the model in validation cohorts containing RA and OA samples, and identified the differential expression trend of the diagnostic biomarkers in validation cohorts containing peripheral blood and synovium samples. Figure 1 exhibits the flow chart for the current study.

## 2 Materials and methods

### 2.1 Dataset collection

In this study, four cohorts containing peripheral blood samples (GSE17755, GSE93272, GSE100191 and GSE205962) and four cohorts containing synovium samples (GSE12021, GSE55235, GSE89408 and GSE39340) were extracted from the GEO database (http://www.ncbi.nlm.nih.gov/geo/). GSE17755 was utilized as a training cohort for screening diagnostic biomarkers, and the rest of the cohorts were used as the validation cohorts. Details of the eight cohorts are shown in Table 1. The Perl software (version 5.30) converted probe names into gene symbols. The average expression value was adopted in case of multiple identical genes appeared in the same expression matrix. The RNA expression values have been log2 (x+1) transformed and normalized. The R software (version 4.3.0) was employed for the subsequent analysis.

**TABLE 1:** The detailed information of the public datasets from GEO.

Dataset	Platform	Tissue	Normal	RA	OA
GSE17755	GPL1291	Peripheral blood	53	112	0
GSE93272	GPL570	Peripheral blood	43	232	0
GSE100191	GPL13497	Peripheral blood	12	7	0
GSE205962	GPL16043	Peripheral blood	4	16	0
GSE12021	GPL96	Synovium	9	12	10
GSE55235	GPL96	Synovium	10	10	10
GSE89408	GPL11154	Synovium	28	152	0
GSE39340	GPL96	Synovium	0	10	7

### 2.2 Identification of differentially expressed autophagy-related genes (DE-ARGs)

Differential expression analysis was performed by the “limma” package (version 3.56.1) with the following threshold: *p*_adj < 0.05 and abs (logFC) > 0.45. Volcano plots and heatmaps of differentially expressed genes (DEGs) were displayed via “ggplot2” (version 3.4.2) and “heatmap” (version 1.0.12) packages. A total of 222 autophagy related-genes were retrieved from the Human Autophagy Database (HADb, http://www.autophagy.lu/) and intersected with DEGs to obtain DE-ARGs. The intersections were presented in a Venn plot drawn by the “VennDiagram” package.

### 2.3 GO and KEGG analysis of DE-ARGs

The “clusterProfiler” package (version 4.8.1) was used to perform Gene Ontology (GO) and Kyoto Encyclopedia of Genes and Genomes (KEGG) enrichment based on the DE-ARGs in the background of org.Hs.eg.db (version 3.17.0). GO analysis included cellular components (CC), molecular function (MF) and biological processes (BP). The GO terms and KEGG pathways with *p*_adj < 0.05 were judged as statistically significant.

### 2.4 Identification of diagnostic biomarkers for RA

Three machine algorithms, including random forest, LASSO, and SVM-RFE, were performed to investigate the significant diagnostic biomarkers for RA. Random forest is an ensemble algorithm that generates multiple decision trees to reach a single decision by aggregating the results of several classifiers (Lee and Park, 2022). It was performed via the “randomForest” package (version 4.7-1.1) with the following parameter: nTree = 500 and the top 10 genes were identified by mean decrease Gini (MDG). LASSO is considered a non-linear variable selection method with the benefit of minimizing the common sum of squared errors (Tian et al., 2019), and the “glmnet” package (version 4.1-7) was used to perform the algorithm. SVM-RFE is generally known for good robustness and stability in determining the optimal variables by removing eigenvectors produced by the SVM (Wang et al., 2022). It was carried out with the “e1071” package (version 1.7-13). The mean misjudgment rates for SVM-RFE were compared with 10-fold cross-validations. Finally, the diagnostic biomarkers were determined by taking the intersection of the genes obtained from the three algorithms.

### 2.5 Establishment and evaluation of nomogram

To evaluate the diagnostic value of diagnostic biomarkers for RA, we utilized the “pROC” package (version 1.18.0) to generate ROC curves and calculate the area under the curve (AUC) with 95% confidence intervals (CI). In addition, the nomogram was generated by the “rms” package. The score corresponding to each gene expression level was displayed on the plot, and the total score was used to evaluate the incidence of RA. When the AUC is above 0.7, the model is considered to have a moderate diagnostic value, while the AUC above 0.9 indicates a high diagnostic value. Besides, the decision curve analysis (DCA) was performed to evaluate the net clinical benefit of the nomogram, and the calibration curve was plotted to demonstrate the discriminatory efficacy of the nomogram for RA.

### 2.6 Immune infiltration and immune-related factors

To evaluate the presence of immune cell infiltration in the microenvironment, we utilized the CIBERSORT algorithm to quantify the relative proportion of 22 immune cells in each sample, and levels of immune cells were visualized by the “ggpubr” package. In addition, Spearman correlation analysis of immune cells with immune factors was performed for the diagnostic biomarkers. Multiple immune factors were retrieved from the TISIDB database (http://cis.hku.hk/TISIDB) (Ru et al., 2019), containing 41 chemokines, 24 immunoinhibtors and 46 immunostimulators (Supplementary Table S1).

## 3 Results

### 3.1 Screening of differentially expressed autophagy-related genes

**FIGURE 2:**
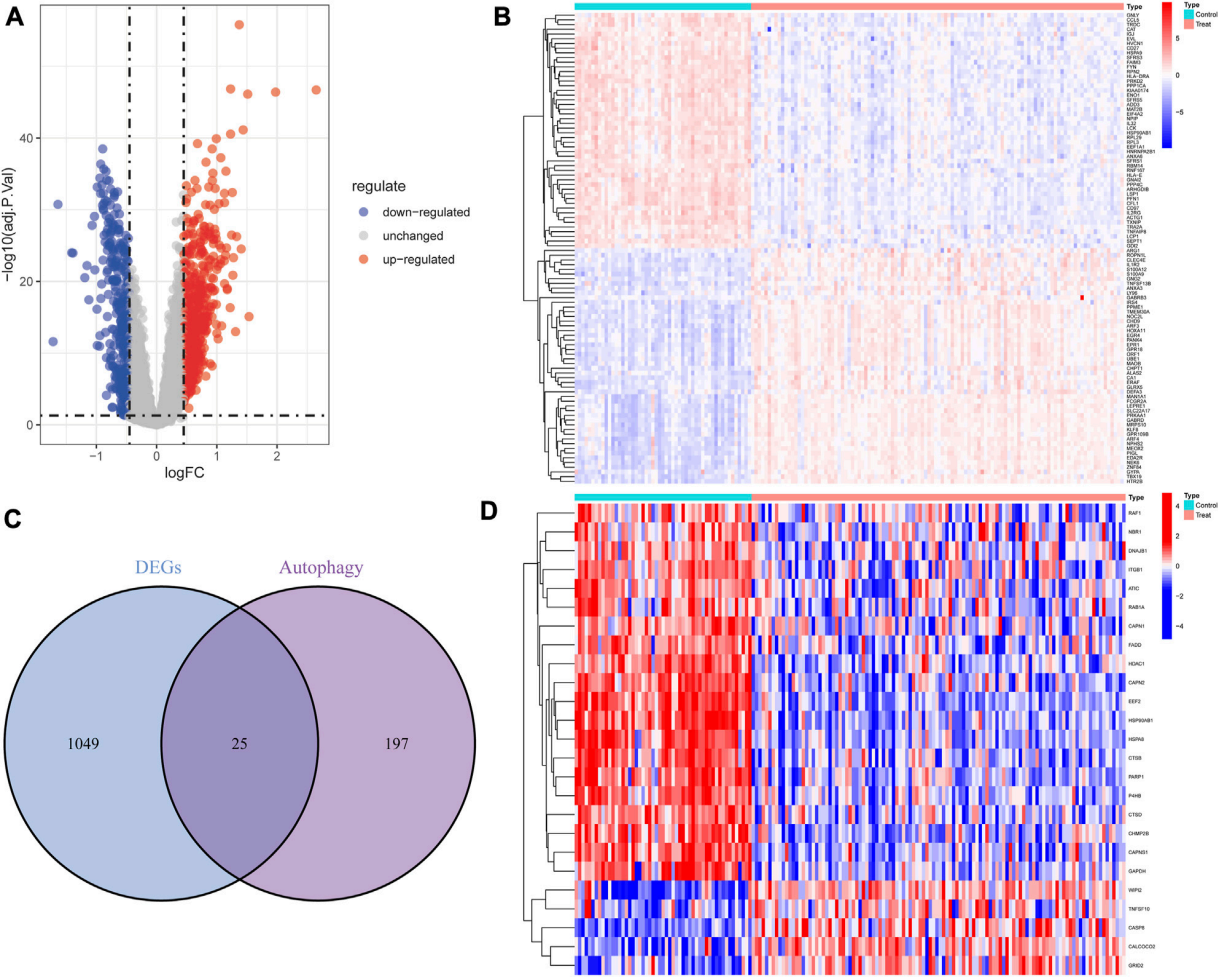
Overview of the differentially expressed autophagy genes in patients with RA and controls. **(A)** Volcano plot of DEGs between RA and controls. Blue nodes represent downregulation, red nodes represent upregulation, and gray nodes represent no significant difference. **(B)** Heat map of DEGs. **(C)** The intersection of DEGs and autophagy-related genes. **(D)** Heat map of 25 autophagy-related DEGs.

Based on the differential analysis, 1,074 DEGs (621 upregulated genes and 453 downregulated genes) were obtained from the peripheral blood samples of GSE17755 and presented by the volcano plot (Figure 2A) and the heatmap (Figure 2B). A total of 25 DE-ARGs (Figure 2C) were obtained by taking the intersection of DEGs and autophagy-related genes, including 20 downregulated genes and 5 upregulated genes (Figure 2D).

### 3.2 GO and KEGG enrichment analysis of DE-ARGs in RA

**FIGURE 3:**
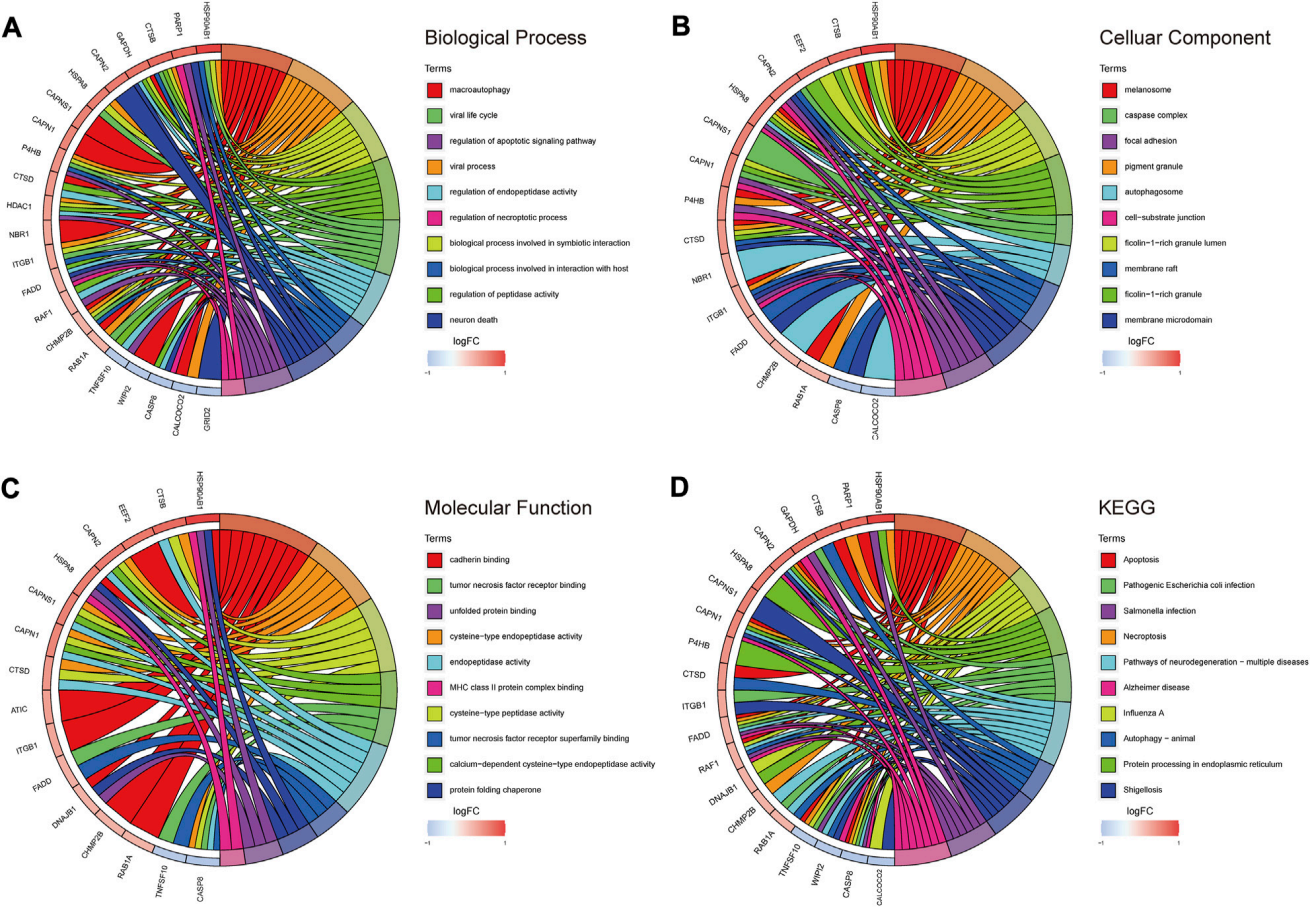
GO/KEGG enrichment analysis of autophagy-related DEGs. **(A)** TOP 10 biological processes pathway. **(B)** TOP 10 cellular component pathway. **(C)** TOP 10 molecular function pathway. **(D)** TOP 10 KEGG pathway.

According to the results of GO analysis, DE-ARGs participated in multiple biological processes in addition to “macroautophagy,” “autophagy-animal” and “autophagosome.” In terms of biological process (BP), DE-ARGs showed a significant association with the “regulation of endopeptidase activity,” “regulation of apoptotic signaling pathway,” and “regulation of necroptotic process” (Figure 3A). DE-ARGs were also involved in several cellular components (CC). CC mainly contained “melanosome,” “caspase complex,” and “focal adhesion” (Figure 3B). Concerning molecular function (MF), “cadherin binding,” “tumor necrosis factor receptor binding,” and “unfolded protein binding” were the main components (Figure 3C). According to the results of KEGG, DE-ARGs were mainly enriched in “apoptosis,” “pathogenic *Escherichia coli* infection” and “*salmonella* infection” (Figure 3D).

### 3.3 Three DE-ARGs served as diagnostic biomarkers for RA

**FIGURE 4:**
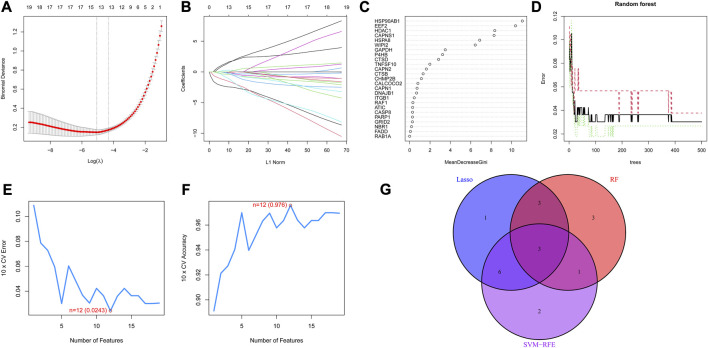
Diagnostic biomarkers obtained through three machine learning algorithms **(A)** Partial likelihood deviance of LASSO logistic regression. **(B)** Coefficient profiles of LASSO logistic regression for 25 DE-ARGs in the 10-fold cross-validation. **(C)** Random Forest algorithm selected DE-ARGs with top 10 MeanDecreaseGini score. **(D)** Relationship between the number of decision trees and the error rate in the Random Forest algorithm. **(E)** Maximum error plots of the SVM-RFE algorithm for selecting DE-ARGs with an error of 0.0243 **(F)** Maximum accuracy plots of the SVM-RFE algorithm for selecting DE-ARGs with an accuracy of 0.976 **(G)** Venn plot demonstrating the overlap of DE-AEGs from the three machine learning algorithms.

Three machine learning algorithms were utilized to screen potential diagnostic biomarkers. Through 10-fold cross-validation in LASSO logistic regression, the penalty parameter was adjusted and ultimately led to the selection of 13 DE-ARGs (Figures 4A, B). The random forest algorithm screened ten genes with the highest MeanReducedGini (Figures 4C, D). Meanwhile, to identify the best feature gene combination, the SVM-RFE algorithm was carried out in screening 12 DE-ARGs (Figures 4E, F). Finally, three autophagy-related diagnostic biomarkers (EEF2, HSP90AB1 and TNFSF10) were obtained for subsequent analysis by overlapping genes from LASSO, SVM-RFE and random forest (Figure 4G).

### 3.4 The performance of the diagnostic model

**FIGURE 5:**
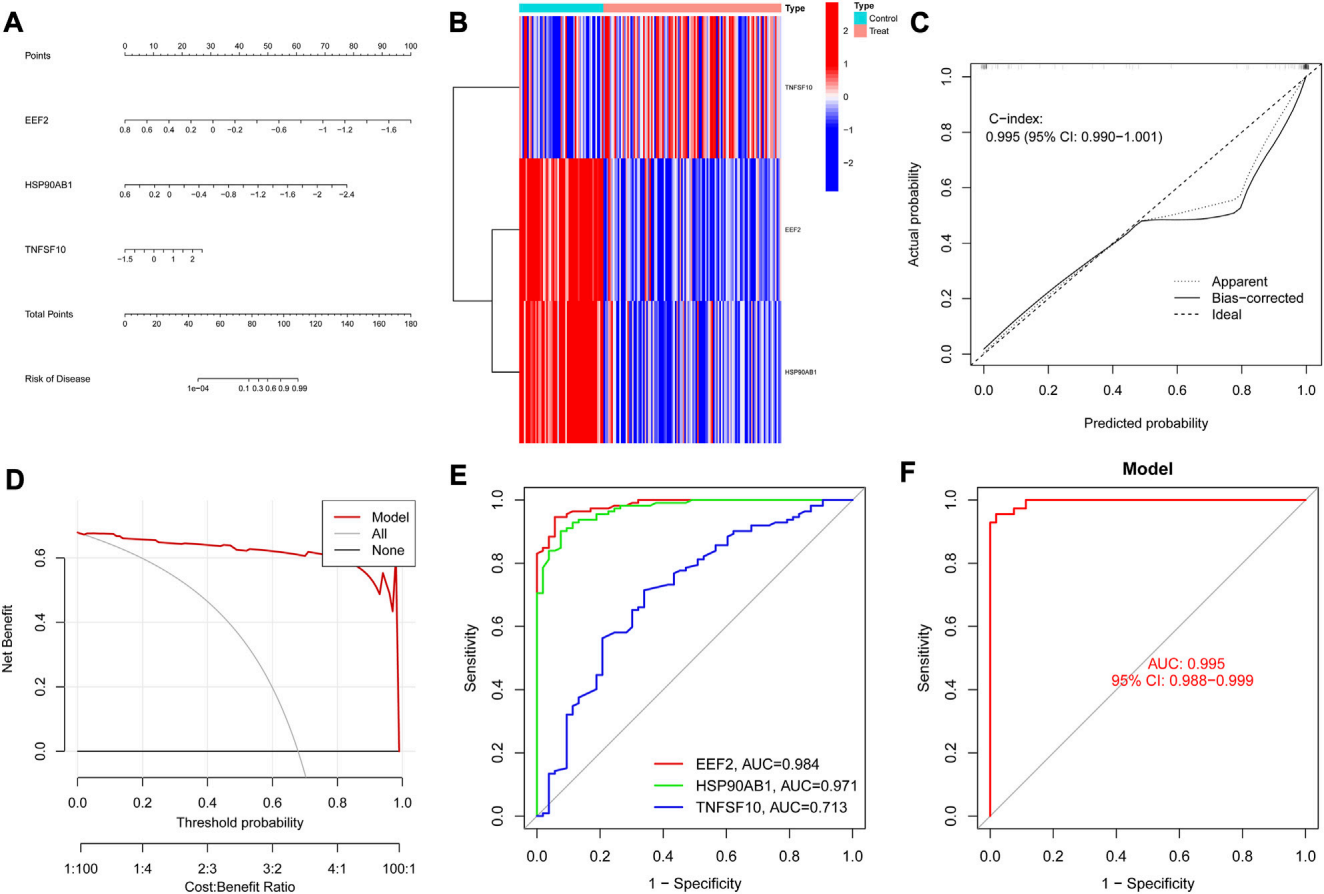
Diagnostic biomarkers efficacy assessment. **(A)** Nomogram of diagnostic biomarkers. **(B)** Heat map of diagnostic biomarkers between RA and controls. **(C)** Calibration curves verify the consistency of the nomogram. **(D)** Decision curve analysis of the diagnostic biomarkers prediction model with values. **(E)** ROC curve for the three genes in the diagnostic prediction model. **(F)** The predictive value of the nomogram in RA from the ROC curve.

As shown in Figure 5A, the nomogram demonstrated the diagnostic value of the model constructed with the three diagnostic biomarkers for RA, and the differential expression levels of the three genes were exhibited in the heatmap (Figure 5B). According to the calibration curve (Figure 5C), the performance of the column line plot closely resembled the ideal model, suggesting that the model had excellent diagnostic accuracy for RA. Furthermore, the curve of the model in the DCA analysis surpassed the two benefit threshold curves, indicating the great efficacy of the model (Figure 5D). According to the results of the ROC curves, the AUC values of EEF2 (0.984), HSP90AB1 (0.971), and TNFSF10 (0.713) were all higher than 0.7 (Figure 5E), and nomogram (AUC = 0.995, 95% CI: 0.988–0.999) exhibited a higher AUC value than each gene (Figure 5F), suggesting that nomogram may possess powerful diagnostic efficacy for RA.

### 3.5 Immune infiltration analysis

**FIGURE 6:**
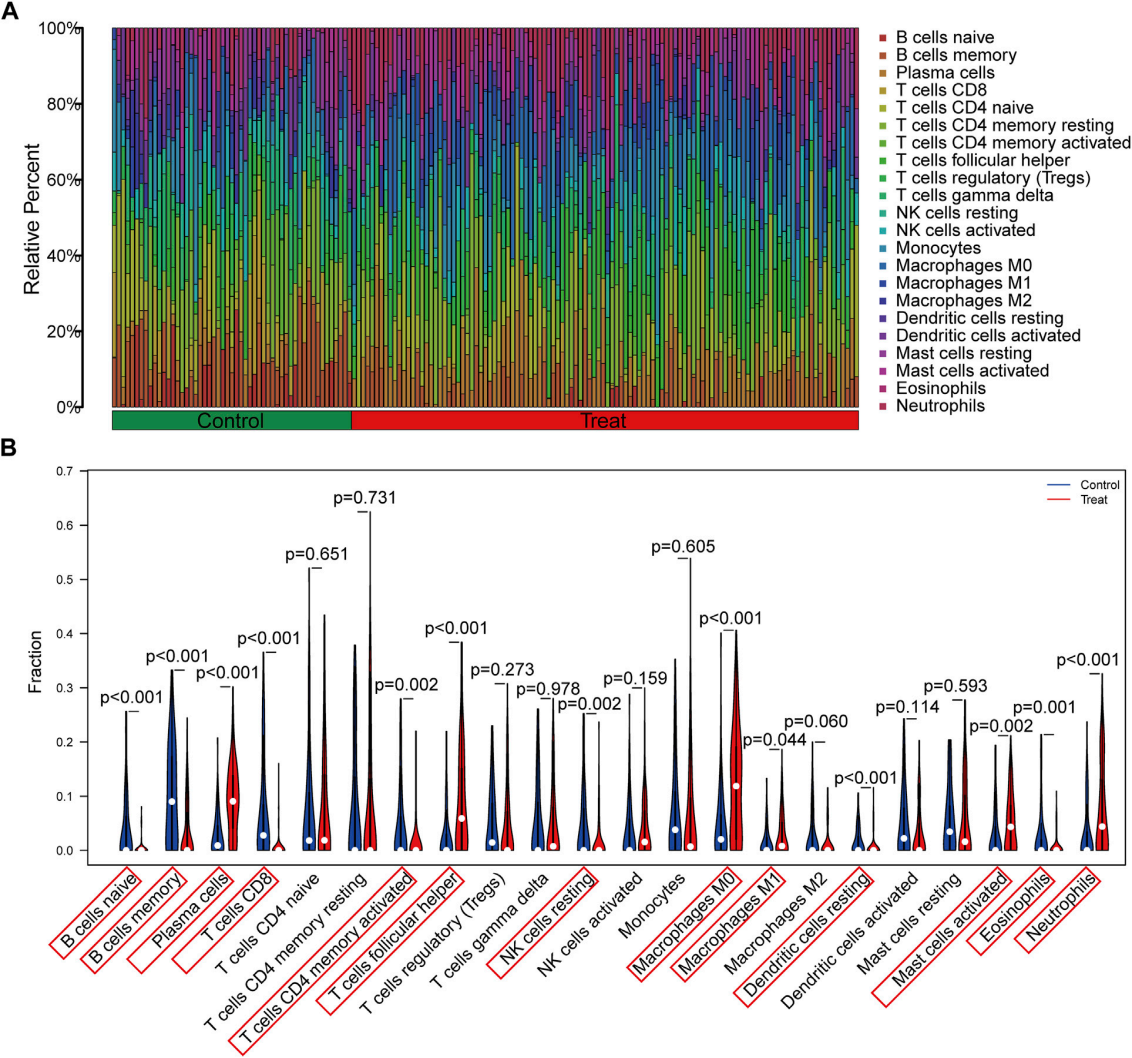
Evaluation and visualization of immune cell infiltration. **(A)** The bar plot shows the proportion of immune cells in different samples. **(B)** Violin diagram indicating 22 types of immune cells.

**FIGURE 7:**
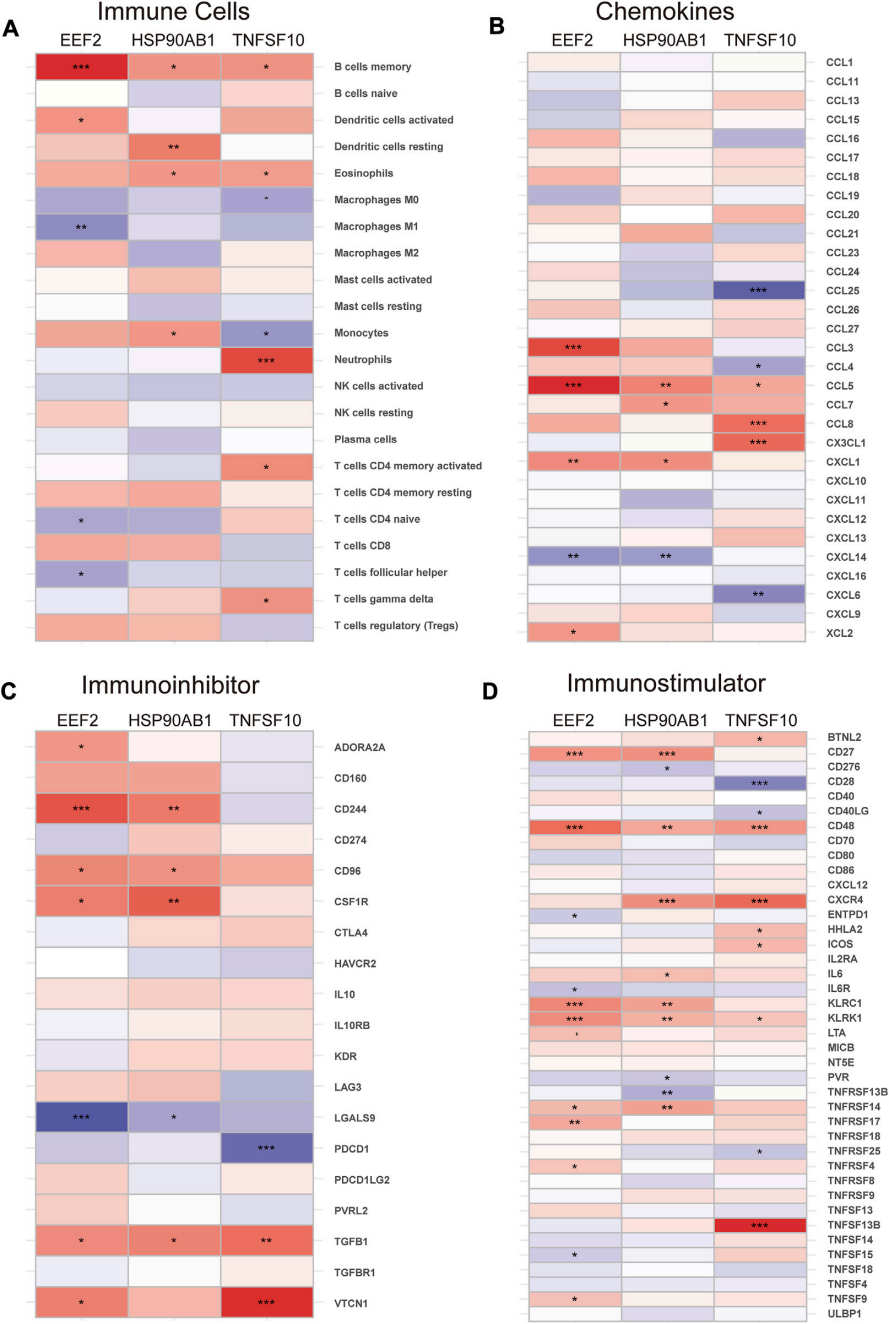
Correlation of diagnostic biomarkers with immune cells and different immune factors. **(A)** Immune cells, **(B)** Chemokines, **(C)** Immunoinhibitors, **(D)** Immunostimulators. Differences between groups are indicated by “*”. *p < 0.05; **p < 0.01; ***p < 0.001.

The immune infiltration analysis in this study was carried out via the CIBERSORT algorithm, and the result was displayed in the histogram (Figure 6A). The violin plots were utilized to compare the immune cell infiltration in RA and normal individuals (Figure 6B). The RA samples exhibited a notable increase in the proportion of plasma cells, T cells follicular helper, macrophages M0, macrophages M1, mast cells activated and neutrophils. In contrast, B cells naive, B cells memory, T cells CD8, T cells CD4 memory activated, NK cells resting, dendritic cells resting and eosinophils showed lower proportions in RA samples. Besides, according to Spearman correlation analysis (Figure 7A), EEF2 showed a negative correlation with macrophage M1 and T cell follicular helper and a positive correlation with B cell memory. HSP90AB1 was positively associated with resting dendritic cells, eosinophils, and B cell memory. TNFSF10 displayed a positive correlation with the T cells CD4 memory activated, neutrophils, eosinophils, and B cells memory, and a negative correlation with macrophages M0. The heatmaps also displayed a notable association between the three diagnostic biomarkers and multiple immune factors, including chemokines (Figure 7B), immunoinhibitors (Figure 7C), and immunostimulators (Figure 7D).

### 3.6 Blood and synovial tissue validation of diagnostic model

**FIGURE 8:**
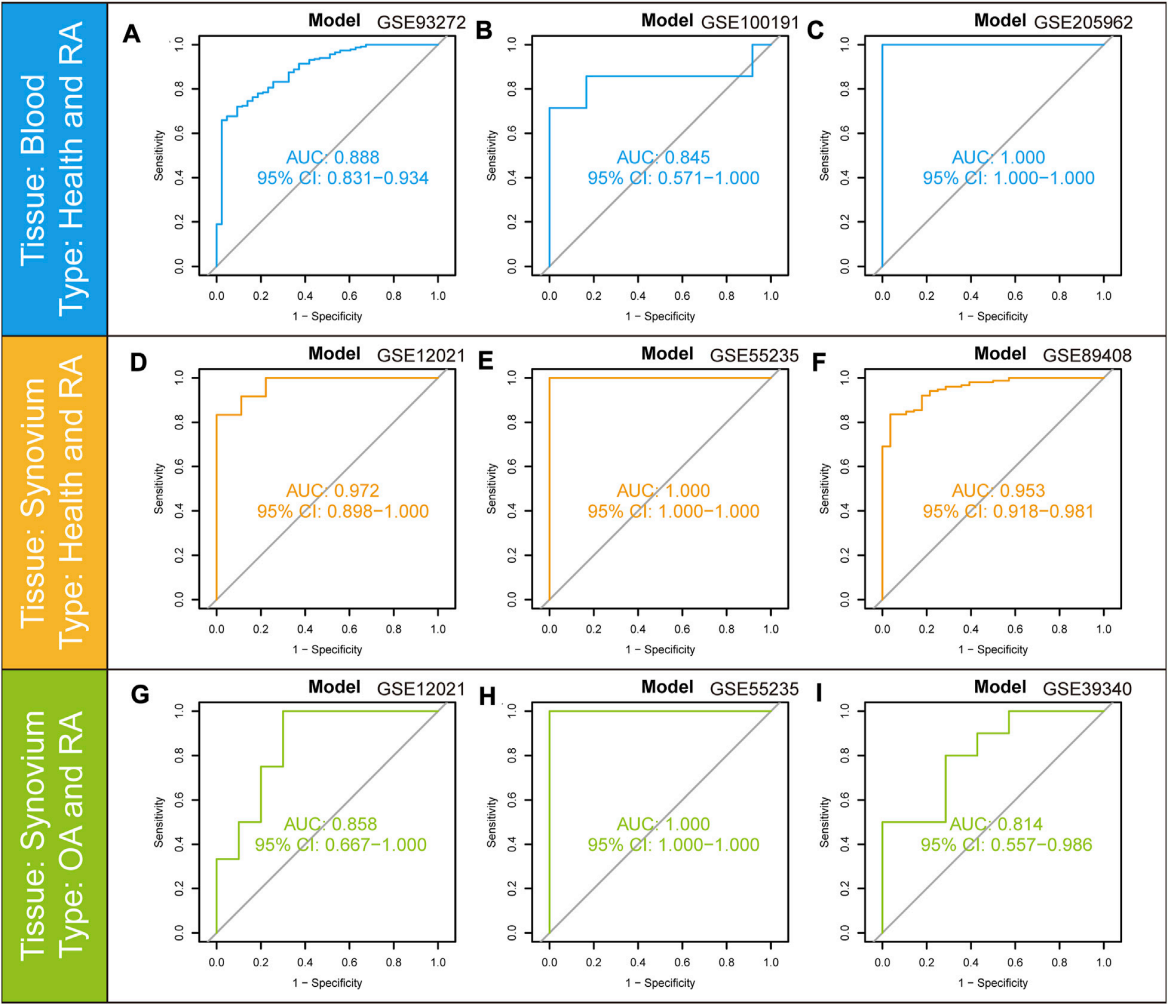
ROC curves and corresponding AUC values for the different expression cohorts. **(A–C)** Blood samples from Control and RA patients in GSE93272, GSE100191, and GSE205962. **(D–F)** Synovium samples from Control and RA patients in GSE12021, GSE55235, and GSE89408. **(G–I)** Synovium samples from OA and RA patients in GSE12021, GSE55235, and GSE39340.

To validate the predictive value of the nomogram model, the ROC curves were further applied to the validation cohorts containing different tissues. The model had solid predictive power in three cohorts containing peripheral blood samples. The AUC value of the model was 0.888 (95% CI: 0.831–0.934) in GSE93272 (Figure 8A), 0.845 (95% CI: 0.571–1.000) in GSE100191 (Figure 8B) and 1.000 (95% CI: 1.000-1.000) in GSE205962 (Figure 8C), respectively, demonstrating exceptional discrimination of the model. The model was further validated in three cohorts containing synovium samples of RA. The AUC value of the model was 0.972 (95% CI: 0.898–1.000) in GSE12021 (Figure 8D), 1.000 (95% CI: 1.000-1.000) in GSE55235 (Figure 8E) and 0.953 (95% CI: 0.918–0.981) in GSE89408 (Figure 8F). We also utilized three cohorts containing synovium samples from osteoarthritis (OA) and RA patients to assess the potential diagnostic value in differentiating RA from OA individuals. The AUC value of the model was 0.858 (95% CI: 0.667–1.000) in GSE12021 (Figure 8G), 1.000 (95% CI: 1.000-1.000) in GSE55235 (Figure 8H) and 0.814 (95% CI: 0.557–0.986) in GSE39340 (Figure 8I). The above results showed that the AUC values for the training and all validation cohorts were higher than 0.7, indicating that the model had good stability and robustness.

### 3.7 Differential expression of diagnostic biomarkers in different RA cohorts

**FIGURE 9:**
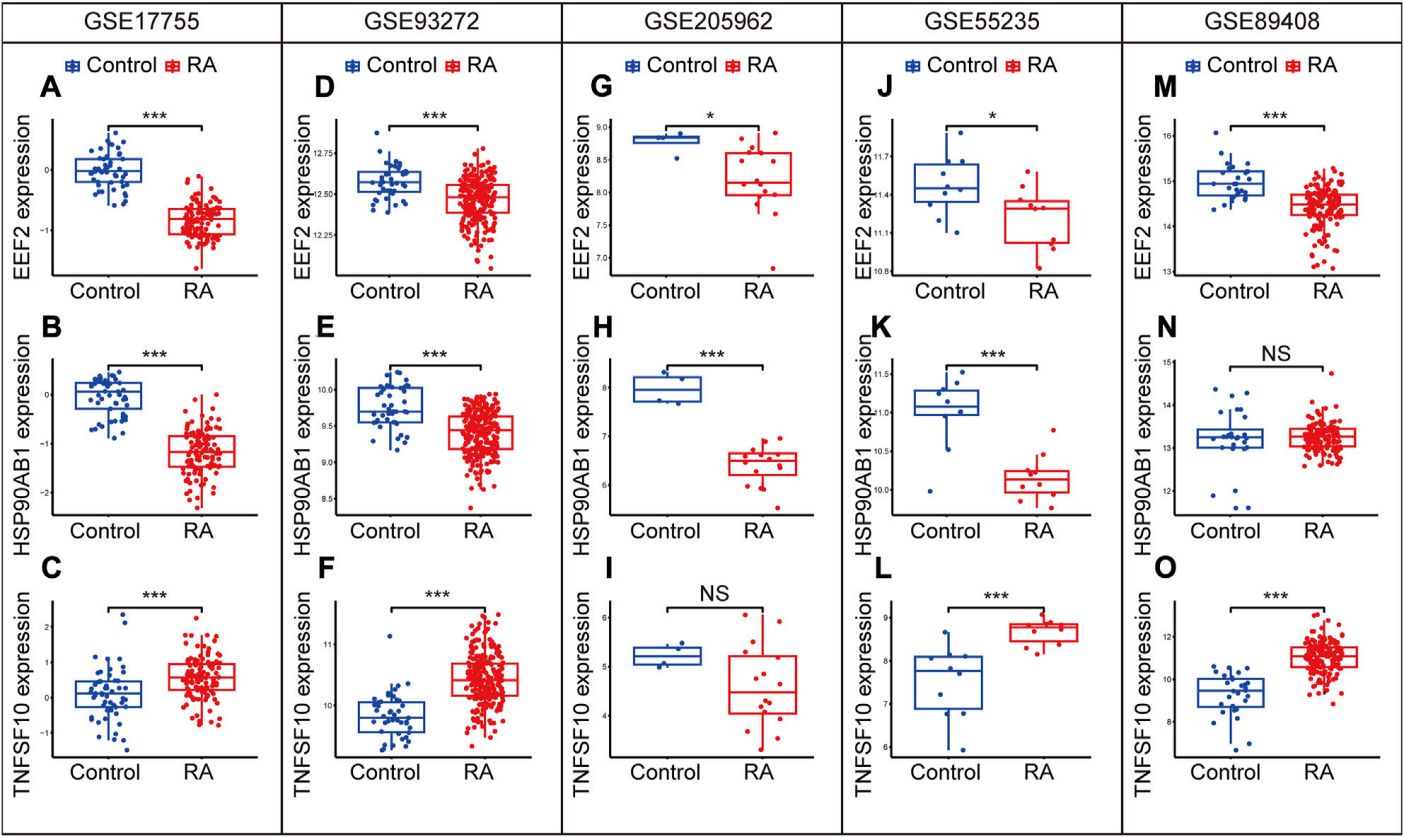
Expression levels of EEF2, HSP90AB1, and TNFSF10 in the GSE17755 **(A–C)**, GSE93272 **(D–F)**, GGSE205962 **(G–I)**, GSE55235 **(J–L)** and GSE89408 **(M–O)**. Differences between groups are indicated by “*”. *p < 0.05; **p < 0.01; ***p < 0.001.

In order to ensure the precision and accuracy of the results, we verified the expression level of diagnostic biomarkers in the cohorts containing peripheral blood (GSE17755, GSE93272 and GSE205962) and synovium samples (GSE55235 and GSE89408). EEF2 was downregulated in the training cohort (Figure 9A) and validation cohorts, including GSE93272 (Figure 9D), GSE205962 (Figure 9G), GSE55235 (Figure 9J) and GSE89408 (Figure 9M). HSP90AB1 was also downregulated in the training cohort (Figure 9B) and validation cohorts, including GSE93272 (Figure 9E), GSE205962 (Figure 9H) and GSE55235 (Figure 9K). However, the difference was not significant in GSE89408 (Figure 9N). Besides, TNFSF10 was upregulated in the training cohort (Figure 9C) and validation cohorts, including GSE93272 (Figure 9F), GSE55235 (Figure 9L) and GSE89408 (Figure 9O). The difference was not significant in GSE205962 (Figure 9I). In summary, the three diagnostic biomarkers in the five cohorts showed the same trend of differential expression.

## 4 Discussion

RA is a common autoimmune disorder with the characteristics of a high disability rate and irreversibility (Tao et al., 2022). It is, therefore, indispensable to determine biomarkers for the early diagnosis of RA. The objective of this study was to identify diagnostic biomarkers associated with RA in the peripheral blood and verify the diagnostic value of DE-ARGs in multiple cohorts containing different tissues. To understand the impact of autophagy on the immune microenvironment of peripheral blood, we also analyzed the correlation between DE-ARGs and immune cells.

Machine learning has developed rapidly in the application of diagnosis. The technology can visualize, understand and classify clinical data, significantly improving the accuracy of medical imaging, biomarkers and other diagnostic methods (Kabade et al., 2021). It has been reported that the machine learning algorithm was created to forecast the transmission of COVID-19, which facilitated virus outbreak control and isolation of infected individuals (John et al., 2022). In this study, 25 DE-ARGs were identified by differential analysis of RA and normal individuals. Three different algorithms were performed to screen for three diagnostic biomarkers (EEF2, HSP90AB1 and TNFSF10). The nomogram model was established based on the three genes and had great predictive value for the training cohort and eight validation cohorts containing peripheral blood, as well as synovium samples. According to immune infiltration analysis, the diagnostic biomarkers had a significant correlation with various immune cells and factors. Furthermore, we also confirmed that the diagnostic biomarkers were remarkably differentially expressed in the five cohorts.

Eukaryotic elongation factor 2 (EEF2) is an elongation factor that enhances translational elongation by mediating translocation (Shen et al., 2021) and can serve as a promoter to accelerate cell proliferation for improved wound healing (Kaleci and Koyuturk, 2020). Previous research has shown that overexpression of EEF2 kinase can improve cellular autophagy by promoting the transformation of microtubule associated protein LC3-I into LC3-II in cells and the formation of acidic vesicle organelle (Hait et al., 2006). CircEEF2 also contributes to autophagy by inhibiting the mTOR pathway and upregulating the expression of autophagy-related gene 5 (ATG5) and ATG7 (Yong et al., 2020). Regarding tissue repair, activation of EEF2 was observed to reduce apoptosis and enhance the recovery of dense connective tissue (Chen et al., 2023) by promoting autophagic flux (Pires Da Silva et al., 2020). Fibroblasts are the critical effector cells in RA (Bartok and Firestein, 2010). EEF2 kinase has been reported to promote cellular autophagy to improve fibroblast differentiation by activating the p38MAPK signaling pathway (Wang et al., 2018). Multiple bioinformatics studies have identified EEF2 as an immune-related biomarker (Melaiu et al., 2012; Lin et al., 2021; Xin et al., 2023). In this study, immune infiltration results revealed a noteworthy correlation between the levels of EEF2 expression and the content of Macrophage M1 and B cell memory. The role of macrophages is crucial in the pathology of RA (Liu and Proud, 2016). The latest research suggests that autophagy activation promotes the transformation of macrophage M1 (pro-inflammatory) into macrophage M2 (anti-inflammatory), which exerts a protective effect against collagen induced arthritis (CIA) (Zhang et al., 2023). A previous study reported that EEF2 kinase might be activated by oxidized low density lipoprotein (oxLDL) through increased calcium ion concentration and contributes to macrophage survival (Chen et al., 2009). Meanwhile, the secretion levels of inflammatory factors in macrophage M1 were significantly altered in EEF2 kinase mutant mice (Liu and Proud, 2016), suggesting that EEF2 may affect RA progression by influencing macrophage polarization. Numerous studies have shown that B cells significantly impact RA from both pathology (Wu H. et al., 2018; Qin et al., 2022) and treatment (Gazeau et al., 2017; Bergantini et al., 2020), whereas evidence about the regulation of EEF2 on B cells is rarely reported, which suggests that further research is necessary to investigate the effects of EEF2 on the immune cells of RA.

Heat shock protein 90 kDA alpha, class B, member 1 (HSP90AB1) is a member of the HSPs family that acts as molecular chaperones to support protein folding and stability maintenance, particularly following exposure to multiple cellular stresses (Haase and Fitze, 2016). HSP90AB1 may inhibit the upregulation of MMP-13 from mitigating transitional degradation of articular cartilage in arthritis (Fan et al., 2009). The loss of endoplasmic reticulum homeostasis is a crucial factor in the progression of autoimmune inflammatory disorders (Miglioranza Scavuzzi and Holoshitz, 2022). One research has revealed that transfection of human granulosa cells with siRNA targeting HSP90AB1 mRNA reduced autophagy and alleviated endoplasmic reticulum stress, while the autophagy inhibitor 3-MA reversed the above process (Wu Y. et al., 2018), implicating a functional relationship between HSP90AB1 and autophagy in RA. The analysis of immune infiltration revealed that the level of HSP90AB1 expression had the highest correlation with dendritic cells. It has been observed that the synovium and peripheral blood of individuals with RA have a high concentration of dendritic cells (Marzaioli et al., 2021), which contribute to the initiation and process of RA by antigen presentation and immune coordination (Wehr et al., 2019). The apoptosis of dendritic cells may reduce T-cell stimulatory capacity, which exerts anti-inflammatory effects in RA (Baldwin et al., 2010). A previous study revealed that HSP90AB1 but not HSP90 alpha was involved in the antiapoptotic response of dendritic cells mediated by CpG-B ODN (Kuo et al., 2007), suggesting inhibition of dendritic cells by HSP90AB1 may serve as a potential therapeutic intervention for RA.

TNFSF10, also known as tumor necrosis factor-related apoptosis-inducing ligand (TRAIL), belongs to the TNF ligand family, which is believed to have a protective impact against RA by regulating systemic inflammatory autoimmune responses. It has been proven that TNFSF10 promotes the proliferation of RA fibroblasts in a dose-dependent manner by activating ERK, p38, and PI3K/Akt pathways (Morel et al., 2005). Administration of human serum albumin (HSA) conjugate linked with TRAIL (HSA-TRAIL) via the tail vein in CIA mice significantly improved morbidity together with clinical scores and reduced serum levels of IL-2, IL-1β, TNF-α, and IFN-γ (Byeon et al., 2014). The current research revealed a correlation between the level of TNFSF10 expression and the content of neutrophils and memory CD4 T cells. Neutrophils are the most abundant circulating white blood cells in humans, and dysregulation of neutrophils is also responsible for the pathogenesis of RA. A clinical study revealed a significant correlation between the content of neutrophils and the duration of morning stiffness in RA patients (Orange et al., 2020). Neutrophil infiltration in the synovium of RA releases IL-17A to stimulate synoviocyte production of CCL20, which attracts monocytes/macrophages to secrete pro-inflammatory factors, including TNF-α and IL-1β, affecting osteoclast differentiation and causing bone destruction (Katayama, 2021). Besides, peripheral blood neutrophils in RA patients secrete higher levels of ROS, disrupting the oxidative/antioxidative balance and exacerbating disease severity (Pradhan et al., 2019). Regarding the treatment of RA, PEGylated TNFSF10 significantly alleviates synovial neutrophil infiltration, cartilage erosion and synovial inflammation in CIA mice (Park et al., 2017). CD4 T cells are served as central effector cells in the persistence of RA. A previous study found a significant correlation between the RA severity and the expression levels of TNFSF10 on CD4 T cells (Bisgin et al., 2010). In recent years, increased cytotoxic CD4 T cell in the peripheral blood of patients with RA has received increasing attention. The production of IL-17 and IFN-γ from CD4 T cells in peripheral blood may directly contribute to the pathogenesis of RA (Park et al., 2014), while TNFSF10 may selectively activate CD4 T cells to promote IFN-γ production (Tsai et al., 2004), indicating that TNFSF10 may be a potential therapeutic target for RA.

In contrast to other bioinformatics studies examining the role of programmed death genes in RA (Xie et al., 2023; Zhou et al., 2023), this study focused on the diagnostic value of autophagy-related genes in RA. In addition, compared to the previous study (Fan et al., 2023), this research utilized three machine learning algorithms to screen diagnostic biomarkers for the construction of the diagnostic model that showed excellent diagnostic efficacy in multiple cohorts containing samples from different tissues and diseases.

## 5 Conclusion

In the present study, we identified three diagnostic biomarkers (EEF2, HSP90AB1 and TNFSF10) by three machine learning algorithms and constructed a nomogram diagnostic model based on the three genes exhibiting great diagnostic value. The diagnostic biomarkers also demonstrated the same trend of differential expression in multiple validation cohorts. Our findings shed essential light on the diagnosis and treatment of RA.

## Data Availability

The original contributions presented in the study are included in the article/Supplementary Material, further inquiries can be directed to the corresponding author.
