# Supplementary material for: Machine learning and bioinformatics analysis to identify autophagy-related biomarkers in peripheral blood for rheumatoid arthritis
Source: Front Genet. 2023 Sep 13;14:1238407. doi: 10.3389/fgene.2023.1238407 (PMC10533932; doi:10.3389/fgene.2023.1238407)
Supplement: Supplementary file 1 [file Table1.DOCX]

https://www.jianguoyun.com/p/DUN41HIQ7N7cCxiM1owFIAA
